# Combined Effects of HLA-B*57/5801 Elite Suppressor CD8+ T Cells and NK Cells on HIV-1 Replication

**DOI:** 10.3389/fcimb.2020.00113

**Published:** 2020-03-20

**Authors:** Megan E. May, Christopher W. Pohlmeyer, Abena K. Kwaa, Madeleine C. Mankowski, Justin R. Bailey, Joel N. Blankson

**Affiliations:** Department of Medicine, Johns Hopkins Medicine, Baltimore, MD, United States

**Keywords:** NK cells, CD8+ T cells, HIV-1, viral replication, viral suppression

## Abstract

Elite controllers or suppressors (ES) are HIV-1 infected individuals who maintain undetectable viral loads without anti-retroviral therapy. The HLA-B*57 allele is overrepresented in ES suggesting a role for HIV-specific CD8+ T cells in immune control. Natural killer (NK) cells also play a role in controlling viral replication, and genetic studies demonstrate that specific combinations of killer cell immunoglobulin-like receptor (KIR) alleles and HLA subtypes including HLA-B*57 correlate with delayed progression to AIDS. While prior studies have shown that both HIV-specific CD8+ T cells and NK cells can inhibit viral replication *in vitro*, the interaction between these two effector cells has not been studied. We performed *in vitro* suppression assays using CD8+ T cells and NK cells from HLA-B*57 ES either alone or in combination with each other. We found no evidence of antagonism or synergy between the CD8+ T cells and NK cells, suggesting that they have independent mechanisms of inhibition *in vitro*. Our data has implications for combined immunotherapy with CD8+ T cells and NK cells in HIV cure strategies.

## Introduction

Elite suppressors represent a model of a functional cure of HIV-1 infection (Walker and Yu, 2013). A substantial percentage of these subjects have protective HLA alleles (Migueles et al., 2000; Pereyra et al., 2010) and many studies have shown that CD8+ T cells in these subjects are effective at inhibiting viral replication (Migueles et al., 2002, 2008; Betts et al., 2006; Saez-Cirion et al., 2007; Hersperger et al., 2010). The role NK cells play in elite control is less clear, but studies have shown that the combination of some protective HLA- Bw4-80I alleles like HLA-B*57/5801 and certain KIR2DS1 and KIR3DL1 alleles confer more protection than either allele alone (Martin et al., 2002, 2007, 2018; Kamya et al., 2011). This suggests that NK cells may also play a role in elite control. We and others have shown that NK cells from some ES can suppress viral replication albeit generally not as effectively as CD8+ T cells (O'Connell et al., 2009; Tomescu et al., 2012; Marras et al., 2013; Walker-Sperling et al., 2017) and it has also been shown that ES have distinct NK cell profiles (Pohlmeyer et al., 2019). NK cells and CD8+ T cells respond to different signals on infected CD4+ T cells. The targeting of different signals by different effector molecules can potentially lead to synergy. However, antagonism between NK cells and CD8+ T cells has been reported for some viral infections (Su et al., 2001; Andrews et al., 2010; Lang et al., 2012; Mitrović et al., 2012). The interaction between these sets of effector cells in HIV infection will be important to understand if they are to be used together in immunotherapy. Thus, we designed experiments to interrogate how viral replication proceeds in the presence of both CD8+ T cells and NK cells. Our results have implications for HIV cure strategies.

## Methods

### Study Subjects

Blood samples from HIV-positive donors were obtained with written informed consent and subsequently handled in accordance with protocols approved by the Johns Hopkins University Institutional Review Board. All ES were African American and treatment naive and maintained undetectable viral loads in the absence of ART. Viremic controller (VC) 10 maintained viral loads below 500 copies/ml in the absence of ART. The clinical characteristics of the subjects are described in Supplementary Table 1.

### Phenotypic Studies

Blood was collected in ACD-containing tubes and incubated at room temperature overnight. The next day whole blood was stained with the following antibody panel for 15 min at 4°C: HLA-DR-PerCP-Cy5.5 and CD4-BV605 from Biolegend, and CD16-FITC, CD56-FITC, CD38-APC, CD8-APC-H7, CD3-PacBlue from BD Biosciences. Stained blood was then incubated at room temperature for 10 min in BD FACS Lysis Buffer at a 1:4 ratio of blood to buffer and then washed three times with PBS before analysis as previously described (Walker-Sperling et al., 2017).

### Suppression Assay

**Figure 1:**
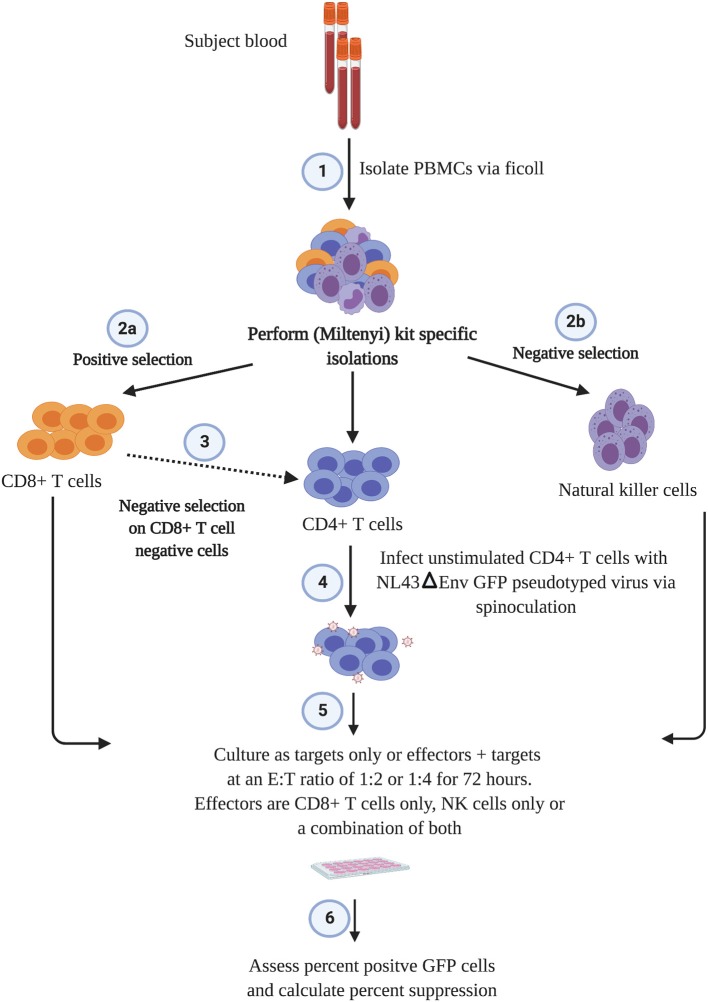
Scheme of the suppression assay used in this study.

**Figure 2:**
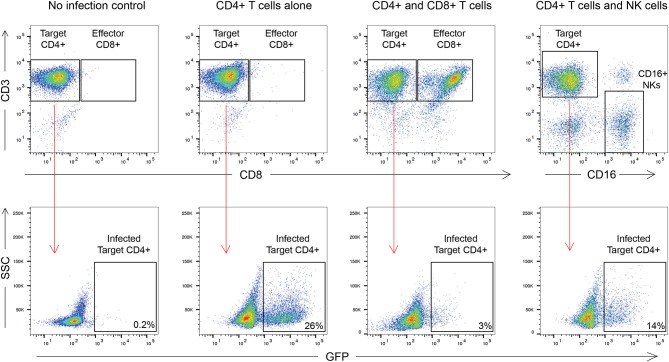
Gating strategy used in this manuscript. CD8+ T cell suppression was calculated as [1–(%GFP + CD4+ T cells cultured with effectors)/(%GFP + CD4+ T cells without effectors)] × 100. For CD8+ T cells this was [1–3/26] × 100 or 88.4% suppression. For NK cells this was [1–14/26] × 100 or 46.2% suppression.

The outline of the suppression assay is shown in Figure 1. Peripheral blood mononuclear cells (PBMCs) were obtained from blood by ficoll centrifugation and NK cells were purified from half of the PBMCs by negative selection with Miltenyi beads. CD8+ T cells were isolated by positive selection with Miltenyi beads from the other half of the PBMCs and CD4+ T cells were isolated from the flow-through cells by negative selection with Miltenyi beads. In all conditions, cells were cultured in RPMI media supplemented with 1% penicillin/streptomycin and 10% fetal bovine serum and 10 U/ml IL-2. The CD4+ T cells were not stimulated to induce immune activation, rather they were infected directly after isolation by spinoculation at 100 ng of HIV-1 p24/100,000 cells with a pseudotyped virus (HIV-1-NL4-3ΔEnv–GFP) for 2 h at 1,200 × g and 37°C. HIV-1-NL4-3ΔEnv–GFP is a lab strain of HIV-1 that has env replaced with gfp. 100,000 CD4+ T cells per well were used for each assay in a 96 well round bottom plate. 25,000 NK or CD8+ T cells per well were added for the 1:4 E:T ratio experiments and 50,000 NK or CD8+ T cells/well were added for the 1:2 E:T ratio. 25,000 NK cells and 25,000 CD8+ T cells were added together for the 1:1:4 E:T ratio experiments. The final volume was 200 ul/ml. After 3 days, cells were harvested and stained with the following panel: CD3 (APC), CD8 (APC-H7), CD16 (PerCP-Cy5.5) and CD56 (PE-Cy7) (all from BD Biosciences). Viable cells were gated based on forward vs. side scatter plots for the majority of these experiments. Because of HIV mediated downregulation of surface CD4, CD4+ T cells were defined as CD3 positive, CD8 negative cells as outlined in Figure 2. GFP expression was used to assess the percentage of infected cells. The percentage of infected CD4+ T cells was typically 5 to10 with a median of 8. The percentage of viral suppression was calculated as [1–(%GFP + CD4+ T cells cultured with effectors)/(%GFP + CD4+ T cells without effectors)] × 100 as previously described (Pohlmeyer et al., 2013; Kwaa et al., 2019).

CD4+ T cells from ES3 were used in transwell experiments. After spinoculation, 1 million infected CD4+ T cells in 1 ml of media was placed in the outer chamber of wells in a 24 well-plate and 500,000 CD8+ T cells or NK cells in 150 ul of media was placed in the transwell for an E:T ratio of 1:2. In control experiments, 1 million infected or uninfected CD4+ T cells were cultured alone in each well or were cultured together with CD8+ T cells or NK cells at the same E:T ratio in 1.5 ml of media without transwells. Staining was performed as outlined above with the addition of Pac-Blue conjugated Annexin V.

### Statistics

The parametric, one way ANOVA with Dunn's multiple comparison test was used to compare the NK cell and CD8+ T cell mediated suppression of viral replication. The Bliss independence model (Bliss, 1939) was used to predict combined suppression of NK cells and CD8+ T cells as previously described (Jilek et al., 2012; Mankowski et al., 2018).

## Results

The suppression assay used here has been previously described for CD8+ T cell and NK cell mediated suppression (Walker-Sperling et al., 2017; Veenhuis et al., 2018; Kwaa et al., 2019). It is based on an assay described by Saez-Cirion et al. (2007) but differs in that the CD4+ T cells are not activated prior to infection. Rather, they are infected directly after isolation with a pseudotyped virus. The absence of an *env* gene means that there is only a single cycle of infection which is very different from the exponential infection that occurs with a replication-competent virus. However, we have seen comparable levels of CD8+ T cell mediated inhibition of cells infected with pseudotyped virus and replication-competent isolates from patients (Veenhuis et al., 2018) and we may have seen even better levels of inhibition if Env epitopes were expressed on infected cells. While this assay does not measure direct killing of infected CD4+ T cells, we and others have shown that direct contact between CD8+ T cells and target CD4+ T cells is needed for suppression (Saez-Cirion et al., 2007; Veenhuis et al., 2018) and we show here that direct contact between ES3 target cells and NK and CD8+ T cells is needed to reduce viral transcription (Supplementary Figure 1). The frequency of Gag and Nef-specific CD8+ T cells in our subjects was not very high (combined median of 2,540 cells/million, Supplementary Table 1) which is consistent with the frequency of total HIV-specific CD8+ T cells found in a prior larger study (Pereyra et al., 2008). However, ES HIV-specific CD8+ T cells have been shown to proliferate in response to antigenic stimulation (Migueles et al., 2002, 2008; Pohlmeyer et al., 2018) and it is likely some level of clonal expansion occurs over the 3 day period of co-culture in our assay. Furthermore, the percentage of infected cells that express antigen is very low initially and therefore the true ratio of effectors to antigen expressing CD4+ T cells at the start of the assay is very high and changes over time as both infection and expansion of HIV-specific CD8+ T proceeds. This is in contrast to typical killing assays that use target cells that are already expressing HIV antigens. Additionally, the much shorter incubation times used in traditional killing assays means there is little chance of proliferation of effector cells so the effector to target ratio stays relatively constant.

**Figure 3:**
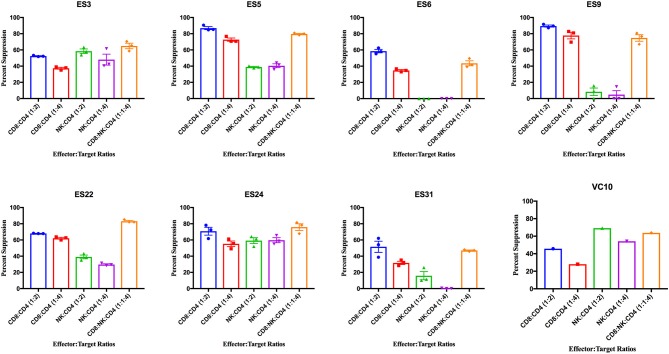
Viral suppression induced by CD8+ T cells, NK cells, and a combination of the two effector cells at the indicated effector to target ratios for each individual. The bar is set to the mean value from 3 replicates per subject with the exception of VC10 where a single replicate was run due to limited numbers of cells. Error bars are the standard error of the mean.

ES CD8+ T cells have been previously shown to have high levels of immune activation (Hunt et al., 2008), and we found higher levels of HLA-DR+CD38- CD8+ T cells and NK cells in ES than in healthy donors in this study (Supplementary Figure 2). CD8+ T cells from all 8 ES inhibited viral replication in autologous CD4+ T cells (Figure 3). This suppressive capacity was specific because very little inhibition was seen when healthy donor CD8+ T cells were cultured with infected autologous CD4+ T cells (Supplementary Figure 3). In contrast, there was significant subject to subject variation of NK cell mediated inhibition of viral infection in both ES (Figure 3) and HDs (Supplementary Figure 3). There was no correlation between the activation status of the NK cells and their suppressive capacity. In subjects that are HLA-B Bw4-80I positive, a correlation between suppressive capacity and KIR3DS1 expression on NK cells has been established (Alter et al., 2007). All the ES in this study are HLA- Bw4-80I positive so we asked whether KIR3DS1 expression could explain the heterogeneous NK cell responses. All 8 subjects were KIR3DS1 negative consistent with the low frequency of this allele in African-Americans (Jiang et al., 2010). Thus, the variable NK cell suppressive responses seen here could not be explained by this allele.

**Figure 4:**
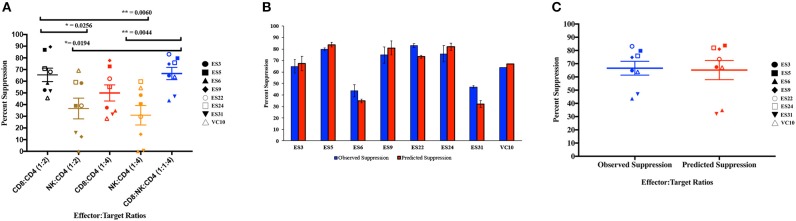
Composite viral suppression by CD8+ T cells, NK cells, and a combination of the two effector cells **(A)**. The mean value for each subject is plotted. A comparison of the observed suppression by the combination of NK cells and CD8+ T cells (blue columns) to the degree of suppression predicted by the Bliss independence model (red columns) is shown for each individual **(B)** and for the cohort **(C)**. The bar is set to the mean value from 3 replicates per subject. Error bars represent the standard deviation from the mean.

As shown in Figure 4A, CD8+ T cell responses at the 1:2 E:T ratio were significantly more effective than NK cell responses at both E:T ratios as previously reported (Walker-Sperling et al., 2017). We combined NK cells and CD8+ T cells in a 1:1:4 ratio with infected autologous CD4+ T cells and found that while there was subject to subject variation, the combination of effector cells was significantly more effective at suppressing viral replication than NK cells alone at both the 1:4 and 1:2 E:T ratios (Figure 4A). In contrast, the difference between inhibition mediated by the combination of effectors cells and inhibition mediated by CD8+ T cells alone was not statistically significant. However, the presence of NK cells did not lead to a decrease in CD8+ T cell mediated suppression in any of the 8 subjects.

We next investigated the interaction between the two effector cells using the Bliss independence model (Bliss, 1939). This model can be used to identify synergy or antagonism between inhibitors under the assumption that the inhibitors have independent binding sites and independent mechanisms of action. In these subjects, the observed level of inhibition by CD8+ T cells and NK cells in combination at a 1:1:4 E:T ratio was not significantly different from the predicted inhibition for the combination calculated using the Bliss independence model equation and observed inhibition by each inhibitor individually at a 1:4 E:T ratio (Figures 4B,C). Thus, the data suggests that at the E:T ratio tested, the two effector cell types inhibited infection by independent mechanisms, and the inhibitory capacities of the two effector cells were neither antagonistic nor synergistic.

## Discussion

This is the first study to look at the direct interaction between the suppressive capacity of NK cells and CD8+ T cells in HIV infection. CD8+ T cell receptors recognize peptides presented on MHC class I molecules whereas NK cells have diverse receptors including the killer-like immunoglobulin receptors that recognize MHC proteins, C-type Lectin-like receptors that recognize stress antigens, and natural cytotoxicity receptors that recognize viral antigens. We asked whether the two different effector cells could work synergistically to inhibit viral replication since they have receptors that recognize different signals on infected CD4+ T cells. In particular, HIV-1 nef (Schwartz et al., 1996) and vpu (Apps et al., 2016) proteins have been shown to downregulate HLA proteins which leads to evasion from CD8+ T cell responses (Collins et al., 1998) but should activate NK cell responses due to the absence of ligands for inhibitory NK cell receptors. The Bliss model was used to formally assess the interaction between the cells and we found no evidence for antagonism or synergy at the E:T ratio we tested. Our study is limited by the relatively small number of subjects studied, the fact that we only looked at the combined effect of the two effector cell types at one E:T ratio, and by unexplained subject to subject variation in NK cell suppressive capacity. Interestingly, NK cells have been shown to enhance CD8+ T cell responses in CMV-infected mice (Robbins et al., 2007). In contrast, other studies have shown that NK cells can inhibit CD8+ T cell responses in LCMV-infected mice (Su et al., 2001; Lang et al., 2012) and CMV-infected mice (Andrews et al., 2010; Mitrović et al., 2012). Our *in vitro* system is incapable of capturing such complicated events that are partially mediated by third party cells. However, even with these limitations, we can conclude that NK cells and CD8+ T cell were not antagonistic at the E:T ratio we analyzed in any of the 8 subjects studied. Independent inhibition by the two effector cell types in combination is promising because HIV-specific CD8+ T cells from chronic progressors do not proliferate effectively in response to antigen (Migueles et al., 2002, 2008) so it might be challenging to induce large numbers of effective cells with immunotherapy. Our data suggest that while NK cells do not work in synergy with CD8+ T cells *in vitro*, they also do not directly antagonize CD8+ T cell mediated inhibition of viral replication. Thus, strategies that employ both types of effector cells for immunotherapy may not result in antagonism, but this will need to be confirmed in larger cohorts of patients and with clinical trials.

## Data Availability Statement

All datasets generated for this study are included in the article/Supplementary Material.

## Ethics Statement

The studies involving human participants were reviewed and approved by Johns Hopkins IRB. The patients/participants provided their written informed consent to participate in this study.

## Author Contributions

MEM, CP, and AK performed the experiments and analyzed data. MCM analyzed data. JRB and JNB supervised the experiments and data analysis and wrote the paper.

### Conflict of Interest

The authors declare that the research was conducted in the absence of any commercial or financial relationships that could be construed as a potential conflict of interest.

## Supplementary Material

Supplementary Figure 1 Effect of transwells on CD8+ T cell and NK cell mediated suppression of viral replication.

Supplementary Figure 2 HLA-DR+CD38+ **(A)** and HLA-DR+CD38- **(B)** expression on ES, healthy donor (HD), and chronic progressor (CP) CD4+ T cells, CD8+ T cells, and NK cells.

Supplementary Figure 3 Suppressive capacity of HD CD8+ T cells and NK cells at E:T ratios of 1:4 and 1:2.

Supplementary Table 1 Clinical characteristics of ES used in this study.
