# Supplementary figures and images for: Combined Effects of HLA-B*57/5801 Elite Suppressor CD8+ T Cells and NK Cells on HIV-1 Replication
Source: Front Cell Infect Microbiol. 2020 Mar 20;10:113. doi: 10.3389/fcimb.2020.00113 (PMC7098910; doi:10.3389/fcimb.2020.00113)

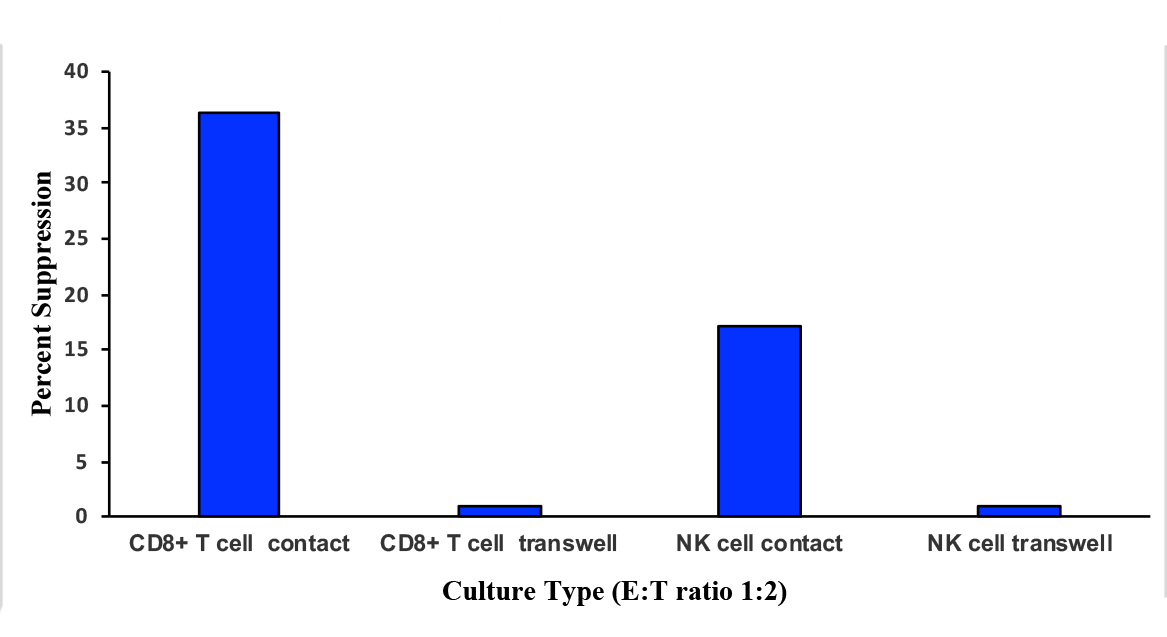

Supplement: Supplementary Figure 1 — Effect of transwells on CD8+ T cell and NK cell mediated suppression of viral replication. [file Image_1.TIF]

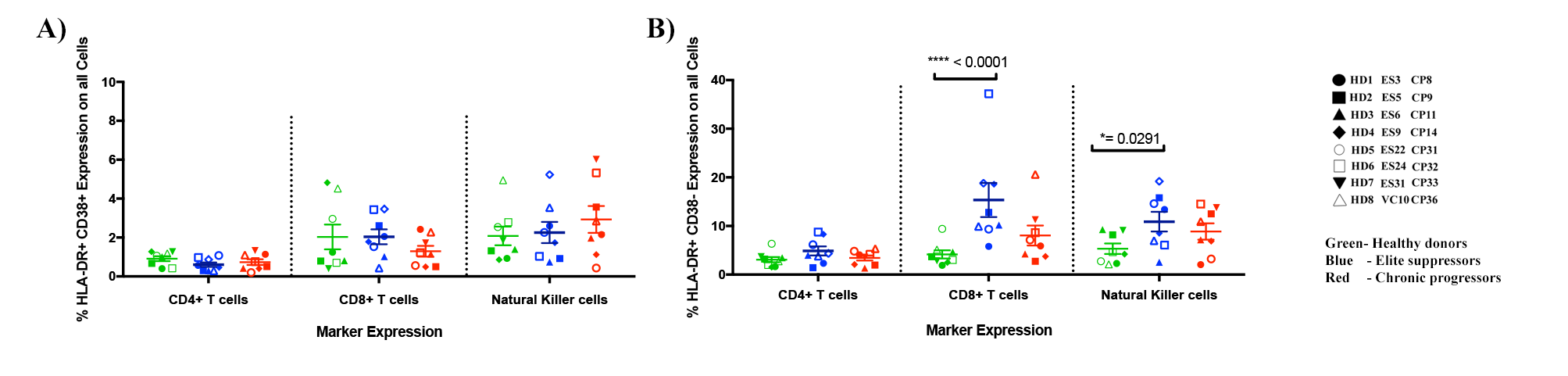

Supplement: Supplementary Figure 2 — HLA-DR+CD38+ (A) and HLA-DR+CD38- (B) expression on ES, healthy donor (HD), and chronic progressor (CP) CD4+ T cells, CD8+ T cells, and NK cells. [file Image_2.TIF]

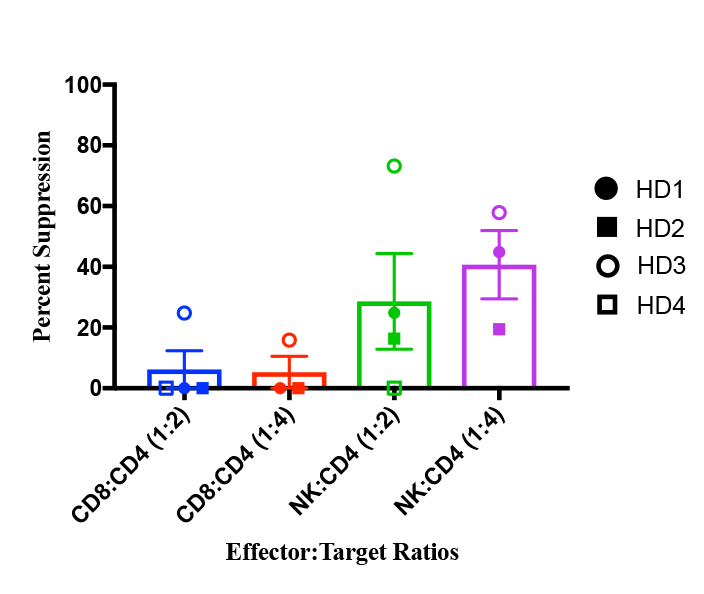

Supplement: Supplementary Figure 3 — Suppressive capacity of HD CD8+ T cells and NK cells at E:T ratios of 1:4 and 1:2. [file Image_3.TIF]
