# Supplementary material for: Combined Effects of HLA-B*57/5801 Elite Suppressor CD8+ T Cells and NK Cells on HIV-1 Replication
Source: Front Cell Infect Microbiol. 2020 Mar 20;10:113. doi: 10.3389/fcimb.2020.00113 (PMC7098910; doi:10.3389/fcimb.2020.00113)
Supplement: Supplementary Table 1 — Clinical characteristics of ES used in this study. [file Table_1.DOCX]

|  | Gender | Years  HIV positive | Viral load  (copies/mL) | CD4 count  (cells/uL) | Protective  HLA allele | Gag-specifc SFC/10^6^ PBMC | Nef-specifc SFC/10^6^ PBMC |  |
| --- | --- | --- | --- | --- | --- | --- | --- | --- |
| ES3 | F | 21 | < 20 | 1046 | HLA-B*57 | 195 | 280 | 475 |
| ES5 | F | 22 | < 20 | 676 | HLA-B*57 | 4700 | 770 | 5470 |
| ES6 | F | 21 | < 20 | 554 | HLA-B*57 | 605 | 3560 | 4165 |
| ES9 | F | 16 | < 20 | 798 | HLA-B*27  HLA-B*57 | 1250 | 560 | 1810 |
| ES22 | M | 7 | < 20 | 1063 | HLA-B*57 | NA | NA |  |
| ES24 | M | 4 | < 20 | 1742 | HLA-B*57 | 2190 | 350 | 2540 |
| ES31 | F | 7 | < 20 | 1037 | HLA-B*27  HLA-B*58 | NA | NA |  |
| VC10 | M | 13 | 383 | 531 | HLA-B*57 | NA | NA |  |
|  |  |  |  |  |  |  |  |  |
